# Assessment of Correlation Between Diabetic Retinopathy and Metabolic Biomarkers Using Artificial Intelligence

**DOI:** 10.1155/jdr/9085827

**Published:** 2026-01-14

**Authors:** Mustafa Aydemir, Ahmet Burak Bilgin, Ramazan Sari, Mehmet Erkan Doğan, Mehmet Bulut, Yusuf Akar

**Affiliations:** ^1^ Division of Endocrinology and Metabolism, Department of Internal Medicine, Akdeniz University, Antalya, Turkey, akdeniz.edu.tr; ^2^ Department of Ophthalmology, Faculty of Medicine, Akdeniz University, Antalya, Turkey, akdeniz.edu.tr; ^3^ Department of Ophthalmology, Anatolia Hospital, Antalya Belek University, Antalya, Turkey

**Keywords:** artificial intelligence, diabetes mellitus, diabetic retinopathy screening, metabolic biomarkers

## Abstract

**Background:**

One of the main causes of blindness in the world, diabetic retinopathy (DR) is a dangerous condition that impairs vision in diabetics. Preventing visual loss requires early recognition of DR and prompt treatments. Artificial intelligence (AI) software combined with nonmydriatic fundus cameras has demonstrated encouraging gains in DR screening effectiveness. However, there are not many studies that systematically compare the diagnostic effectiveness of various nonmydriatic cameras and AI software in the field of endocrinology, where managing diabetes and its complications is crucial. By offering vital information for enhancing diabetes care plans and fortifying preventative actions in the context of endocrine health, this study seeks to close this knowledge gap.

**Methods:**

This clinical study was conducted at the Akdeniz University endocrinology clinic with 900 volunteer patients who had previously been diagnosed with diabetes but had undiagnosed DR. Fundus images of each patient were captured using three different nonmydriatic fundus cameras. These images were then assessed for varying degrees of DR, ranging from mild to more severe forms, including vtDR and clinically significant diabetic macular edema, utilizing EyeCheckup AI software. Additionally, patients underwent pupil dilation for wide‐angle fundus photography, resulting in four distinct wide‐angle images being taken. Three retina specialists evaluated these four wide‐field fundus images based on the DR treatment guidelines set forth by the American Academy of Ophthalmology. The effectiveness of the AI in detecting DR was determined through statistical analysis, comparing the diagnoses made by the physicians with those provided by the AI. Furthermore, patients filled out a questionnaire regarding their medical history and underwent a lipid panel blood test along with urine tests. These assessments included various metabolic measurements such as HbA1c levels, diabetes duration, low‐density lipoprotein (LDL) cholesterol, high‐density lipoprotein (HDL) cholesterol, triglycerides, urinary albumin levels, glomerular filtration rate (GFR), creatinine, and C‐reactive protein (CRP) levels.

**Results:**

Our study revealed a significant association between the prevalence of DR and diabetes duration, HbA1c, CRP, and urinary albumin levels. The *p* values of this association were 0.000, 0.000, 0.003, and 0.002, respectively. It was also noted that there may be an association between triglyceride levels and DR prevalence; the *p* value of this association was 0.079, with more data needed to establish a strong link. The study also revealed that AI performed satisfactorily in detecting DR from fundus images. The sensitivity and specificity of the different cameras used are as follows: Canon CR2 AF: 95.65% sensitivity, 95.92% specificity; Topcon TRC‐NW400: 95.19% sensitivity, 96.46% specificity; and Optomed Aurora: 90.48% sensitivity, 97.21% specificity.

**Conclusion:**

Our research has shown a significant association between the prevalence of DR and elevated levels of HbA1c, CRP, and urinary albumin and duration of diabetes. This suggests that these biomarkers may serve as valuable predictive indicators in assessing the likelihood of DR. Consequently, the inclusion of these parameters in routine clinical assessments could improve proactive screening strategies, thus enabling early detection and intervention of DR. This, in turn, could reduce the risk of vision loss in affected patients. The study also demonstrates the potential of nonmydriatic fundus cameras used in combination with AI software to detect DR at an early stage.

**Trial Registration:**

ClinicalTrials.gov identifier: NCT04805541

## 1. Introductıon

The most common side effect of diabetes is diabetic retinopathy (DR), which is a major cause of blindness and vision impairment and a major worldwide health concern [1]. To diagnose DR, a thorough retinal examination is necessary. Proliferative DR and diabetic macular edema (DME) complications can cause blindness and significant visual loss. However, successful prevention or management of these consequences depends on early identification. Less than half of diabetic patients adhere to the suggested schedule for routine eye exams, despite the fact that these examinations are essential for preventing vision loss [2].

According to the 2013 TURDEP‐II study, a comprehensive survey carried out in Turkey, the prevalence of diabetes mellitus (DM) was 16.5%, meaning that 6.5 million persons in the country suffer from DM. The frequency of DM has increased by 3% since the TURDEP‐I research, which was carried out 12 years ago [3]. It is currently believed that 20% of Turkish people have diabetes. Based on the most thorough nationwide surveys conducted to date, these results demonstrate that DM is one of the most urgent and pervasive public health issues in Turkey. About 25%–30% of DM patients get DR in one form or another, with 3% developing a kind that could endanger their eyesight [4]. Regretfully, it is difficult to guarantee routine retinal tests for these patients due to Turkey′s shortage of ophthalmologists and retinal specialists.

To improve adherence to retinal examinations required for diagnosing and staging DR, many countries have begun implementing nationwide screening programs using telemedicine [5]. However, establishing a systematic DR screening program faces administrative and budgetary challenges, particularly in low‐ and middle‐income countries like Turkey. Additionally, the large population size further complicates the implementation of a comprehensive screening program. Therefore, there is an urgent need for a low‐cost screening method capable of assessing DR risk in large diabetic populations.

The primary objective of DR screening in individuals with diabetes is to ensure the timely referral of high‐risk patients to an ophthalmologist. Recent studies have highlighted artificial intelligence (AI) algorithms, particularly those utilizing nonmydriatic posterior pole imaging, as a promising solution for this purpose. Research conducted in various countries has demonstrated the effectiveness of AI‐based algorithms in diagnosing DR [6, 7]. Although the standard diagnosis of DR relies on retinal examinations, easily accessible metabolic markers—such as HbA1c, cholesterol levels, and kidney function indicators—show potential for noninvasive prediction of the disease and monitoring its progression [8, 9]. These advancements offer a complementary approach to traditional methods, potentially enhancing the efficiency and accessibility of DR screening programs. Though studies suggest potential links between these markers and DR, their individual diagnostic accuracy remains limited. A comprehensive evaluation of these markers, especially in combination, is essential to develop effective, accessible tools for early DR detection, disease progression prediction, and optimized treatment strategies to improve patient outcomes.

## 2. Material and Methods

### 2.1. Patıents

The study included a total of 900 participants who applied to the Endocrinology and Metabolic Diseases Department at Akdeniz University, had a diagnosis of DM but no known diagnosis of DR. The study was approved by the ethics committee of the Republic of Turkey′s Ministry of Health at Akdeniz University.

The following were the requirements for patients to be included in the study:

1. Patients diagnosed with DM and under the care of the endocrinology department.
2. Age over 18 years
3. No previous diagnosis of DR.
4. No history of laser photocoagulation, intravitreal injections, or surgical interventions related to DR.
5. No history of ophthalmic procedures, such as cataract surgery.
6. Completion and signing of the informed consent form.
7. Absence of any media opacity (e.g., cataracts or other conditions) that could affect the visibility of the retina and optic disc in retinal photographs.

### 2.2. Methods

Patients were recruited for the study at the endocrinology clinic, where they provided signed informed consent to participate. Before the patients undergo ophthalmological examination, various measurements were collected from physical exams, as well as blood and urine tests, including: weight, height, BMI, duration of diabetes, glycated hemoglobin, LDL cholesterol, HDL cholesterol, triglycerides (TG), urine albumin, GFR, creatinine, and CRP levels. Next, three distinct nonmydriatic fundus cameras—the Canon CR2 AF, Topcon TRC‐NW400, and Optomed Aurora—were used to take pictures of the posterior pole. Two wide‐field images were taken of each eye, one focused on the optic disc (Figure 1b) and the other on the macula (Figure 1a).

Figure 1Fundus images taken from patients. (a) Nonmydriatic fundus images centered on the macula from three different cameras. (b) Nonmydriatic fundus images centered on the optic disc from three different cameras.

**Figure:**
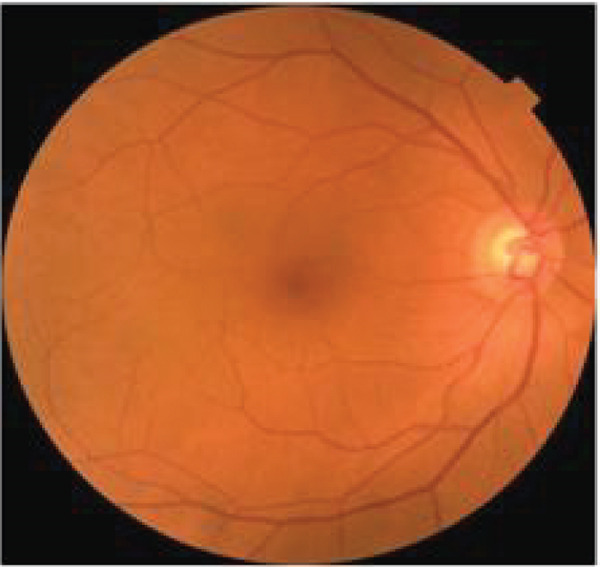
(a)

**Figure:**
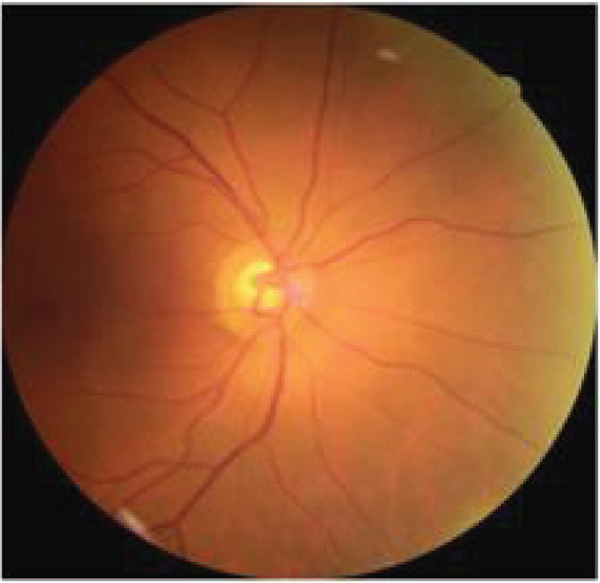
(b)

**Figure 2:**
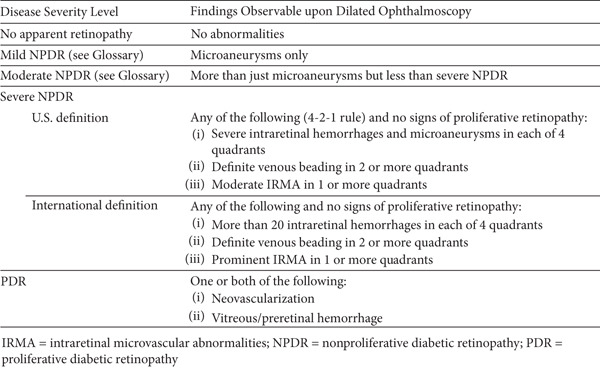
The severity scale for diabetic retinopathy (DR) is based on the Diabetic Retinopathy Preferred Practice Pattern guidelines established by the American Academy of Ophthalmology.

The captured images were uploaded to the EyeCheckup client software and analyzed by an AI system to detect the presence of DR. Based on the pathological findings identified by the AI, patients were classified into one of the following categories, as recommended by the American Academy of Ophthalmology (AAO) [10]: no DR, mild nonproliferative DR (NPDR), moderate NPDR, severe NPDR, or proliferative DR (PDR). This grading process is illustrated in Figure 2.

If a patient′s DR severity was greater than mild nonproliferative DR (NPDR), they were classified as having “more than mild DR (mtmDR).” Patients with severe NPDR or PDR were categorized as having “vision‐threatening DR (vtDR)” [6]. The mtmDR group includes patients who should be referred to an ophthalmologist within 6 months, whereas the vtDR group consists of patients at high risk of significant vision loss, requiring urgent referral (within 1–2 months).

Historically, based on the Early Treatment Diabetic Retinopathy Study (ETDRS) [11], clinically significant diabetic macular edema (CSDME) is defined by the following criteria:

1. Retinal thickening at or within 500 *μ*m of the foveal center
2. Hard exudates at or within 500 *μ*m of the foveal center, if accompanied by adjacent retinal thickening.

According to the AAO Diabetic Retinopathy Preferred Practice Patterns, the presence of hard exudates in the macula is considered as an indicator of suspected CSDME [12]. Patients suspected of having CSDME were also classified under the vtDR category.

**Figure 3:**
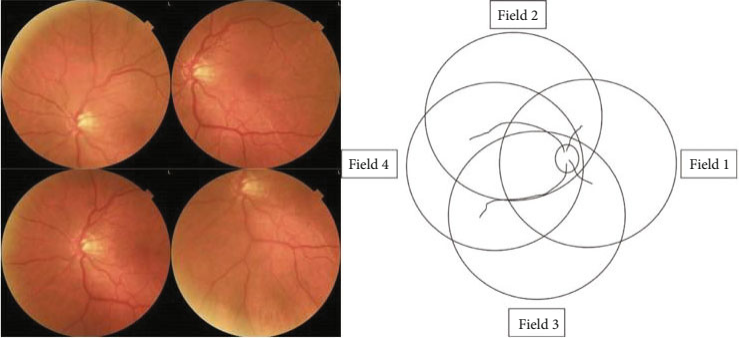
Four wide field imaging.

Each participant underwent pupil dilation, and in addition to the two wide‐field images per eye initially captured for EyeCheckup AI evaluation, an additional set of four 45‐degree quadrant fundus images (Figure 3) was taken using the Canon CR2 AF 45‐degree fundus cameras. These images, which provided coverage of the peripheral retina, along with the original two nonmydriatic images, were meticulously reviewed by three retina specialists. A consensus diagnosis was established for each eye, serving as the definitive or “ground truth” diagnosis for each patient. Classification at the patient level was determined based on the more severely affected eye. The diagnoses generated by EyeCheckup AI were then compared with this ground truth, and statistical analyses were performed to validate the diagnostic accuracy of the AI system.

### 2.3. EyeCheckup AI

In adults (ages 18 and older) with diabetes who have not previously been diagnosed with DR, EyeCheckup is designed to automatically detect mtmDR and vtDR (e.g., severe NPDR, PDR, and/or DME).

**Figure 4:**
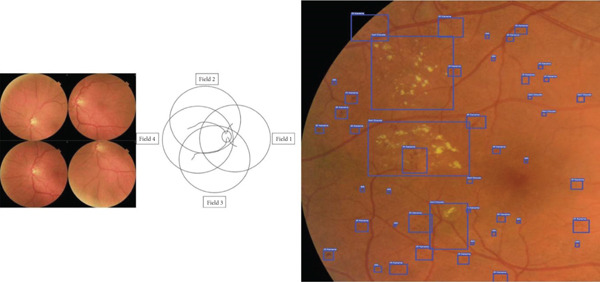
Fundus image labeled with doctors says software and four wide field imaging.

EyeCheckup is optimized to work with Canon CR‐2 AF, Topcon TRC‐NW400, and Optomed Aurora fundus cameras and was created for usage with retinal fundus images. Additionally, the technology displays the anomalies that have been noticed, enabling physicians to see how DR was discovered (Figure 4).

### 2.4. Statıstıcal Analysıs

Endpoints for sensitivity and specificity were developed to evaluate the efficacy of AI‐based diagnosis in medical contexts. The number of correctly recognized positive instances is known as true positives (TP), and the number of correctly identified negative cases is known as true negatives (TN). False negatives (FN) are positive cases that are mistakenly classified as negative, whereas false positives (FP) are negative cases that are mistakenly categorized as positive. Sensitivity (also known as true positive rate) is the probability of a positive test result if the case is in fact positive. It is computed as follows: *s* *e* *n* *s* *i* *t* *i* *v* *i* *t* *y* = *T* *P*/(*T* *P* + *F* *N*). The probability of a negative test result when the case is actually negative is represented by specificity (also known as true negative rate), which is computed as *s* *p* *e* *c* *i* *f* *i* *c* *i* *t* *y* = *T* *N*/(*T* *N* + *F* *P*).

For every type of camera, a diagnosability measure was also computed. The ratio of patients whose images were good enough for EyeCheckup to analyze to those whose images were good enough to be evaluated using the clinical reference standard is known as diagnosability. Lastly, it was determined what proportion of patients needed pupil dilation in order to obtain a sufficient image quality for EyeCheckup. The evaluations of a panel of three retina specialists were then contrasted with the AI‐based diagnosis.

Patients were categorized into subgroups according to various metabolic parameters, such as BMI, duration of diabetes, HbA1c levels, LDL cholesterol, HDL cholesterol, TG, urine albumin, GFR, creatinine, and CRP levels. For example, based on diabetes duration, patients were divided into three groups: those with diabetes for less than 10 years, 10–20 years, and more than 20 years. The prevalence of DR was then assessed within each subgroup to explore potential relationships between these biomarkers and the occurrence of DR. The data were visualized on a graph, and regression analysis was performed to evaluate the strength of the correlation between each biomarker and DR prevalence. From this analysis, *R* ^2^ and *p* values were derived, with a *p* value of less than 0.05 considered statistically significant. Additionally, an *R* ^2^ value exceeding 0.75 was regarded as indicative of a strong correlation between the metabolic biomarkers and the prevalence of DR.

### 2.5. Study Demographics

The study initially enrolled 900 patients from the endocrinology department at Akdeniz University Hospital in Antalya, Turkey. After applying predefined exclusion criteria, 35 patients were excluded, and fundus photographs from the remaining 865 patients were analyzed. The study cohort comprised 900 participants, with a nearly balanced gender distribution of 50.89% male and 49.11% female. The majority of participants had Type 2 diabetes (98.44%), whereas a small proportion (1.56%) had Type 1 diabetes. On average, participants had been living with diabetes for approximately 9.78 years, and their mean age was 58 years. The average weight was 81.52 kg, and the average height was 165.28 cm, resulting in a mean BMI of 29.87. Overall, 3.89% of patients were deemed ineligible for the study, leaving 96.11% eligible for inclusion (Table 1).

**Table 1:** Study population characteristics for diabetic retinopathy screening trial.

**Study population demographics**	
**Total study population**	**900**
Average age in years	58.33 ± 11.16
Male (%)	458 (50.89%)
Female (%)	442 (49.11%)
Average BMI	29.87 ± 5.37
Diabetes type 1 (%)	14 (1.56%)
Diabetes type 2 (%)	886 (98.44%)
Average diabetes duration in years	9.78 ± 8.07
Weight in kilograms	81.52 ± 15.40
Height in centimeters	165.28 ± 15.70
Diabetic retinopathy negative	721
Diabetic retinopathy positive	144
Diabetic retinopathy prevalence (%)	16.65%
Hba1c (%)	7.60 ± 1.71
LDL cholesterol in mg/dL	102.60 ± 33.52
HDL cholesterol in mg/dL	47.03 ± 13.37
Triglycerides in mg/dL	276.44 ± 97.30
Urine albumin *μ*mg/dL	105.44 ± 95.56
GFR in mL/min	37.54 ± 20.30
Creatine in mg/dL	0.920 ± 2.85
CRP in mg/L	2.66 ± 4.83
Eligible patients (%)	865 (96.11%)
Ineligible patients (%)	35 (3.89%)

### 2.6. Determination of Sample Size

The minimum sample size required for the study was calculated with the validation of the AI diagnostic software in mind. Based on the assumption that the AI algorithm would achieve a diagnostic accuracy rate exceeding 90%, and accounting for a 5% Type‐I error rate, 80% statistical power, and varying effect sizes, the minimum sample size was determined to be 778 participants [13]. A 10% data loss was expected due to ineligible patients. That is the reason a minimum of 855 patients was recruited for this study [13].

## 3. Results

### 3.1. Statistical Results

#### 3.1.1. Correlation Between Prevalence of DR and Metabolic Biomarkers

Table 2 shows correlation between different metabolic biomarkers markers and the prevalence of DR. The study found significant correlation between the prevalence for DR and the duration of diabetes, HbA1c, CRP and urine albumin levels. The *p* values of data being 0.000, 0.000, 0003, and 0.002 when the relationship between diabetes duration/HbA1c/urine albumin/CRP levels and the prevalence of diabetes were assessed, respectively. The study also indicated the possibility of a relationship between TG and prevalence diabetes retinopathy with *p* value being 0.079, yet further data are needed to indicate a strong relationship. Figure 5 shows significantly correlated biomarkers plotted against the prevalence of DR and their respective *R* ^2^ values.

**Table 2:** Prevalence of DR against metabolic markers.

	**Number of patients**	**Prevalence (any DR)**	**DR AI** +	**DR AI** −	**DR ground truth (three retina specialists)** +	**DR ground truth (three retina specialists)** −	**P** **value**
Diabetes duration							
0–10 years	496	9.32%	56	427	45	438	0.001
10–20 years	298	22.30%	62	225	64	223	
20+ years	106	36.84%	34	61	35	60	
Hba1c							
3–6	93	3.33%	4	86	3	87	0.001
6–9	666	15.49%	103	536	99	540	
9–12	115	31.82%	39	71	35	75	
12+	26	26.92%	6	20	7	19	
Urine albumin							
0–20	630	14.59%	95	515	89	521	0.002
20–200	217	18.93%	40	166	39	167	
200+	53	32.65%	17	32	16	33	
Urine albumin/creatinine ratio							
0–20	634	15.47%	100	514	95	519	0.14
20+	266	19.52%	52	199	49	202	
GFR							
60+	814	16.60%	136	647	130	653	0.68
30–60	72	18.84%	14	55	13	56	
30–15	8	14.29%	1	6	1	6	
< 15	6	0.00%	1	5	0	6	
LDL cholesterol							
0–100	495	15.86%	75	398	75	398	0.48
100–200	395	17.23%	73	310	66	317	
200+	10	33.33%	4	5	3	6	
HDL cholesterol							
0–50	639	16.78%	109	505	103	511	0.87
50+	261	16.33%	43	208	41	210	
Triglyceride							
0–150	549	16.45%	90	439	87	442	0.07
150–500	340	16.21%	59	268	53	274	
500+	11	44.44%	3	6	4	5	
CRP							
0–5	794	15.22%	123	639	116	646	0.002
5+	106	27.18%	29	74	28	75	
All patients	900	16.65%	152	713	144	721	

Figure 5The figure illustrates the relationship between the prevalence of any diabetic retinopathy (Any DR) and diabetes duration, HbA1c, urine albumin, and CRP levels.

**Figure:**
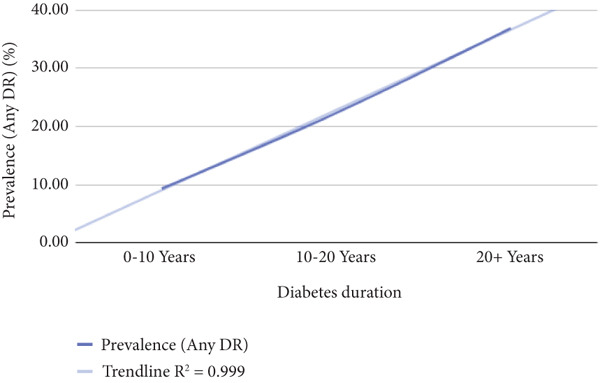
(a)

**Figure:**
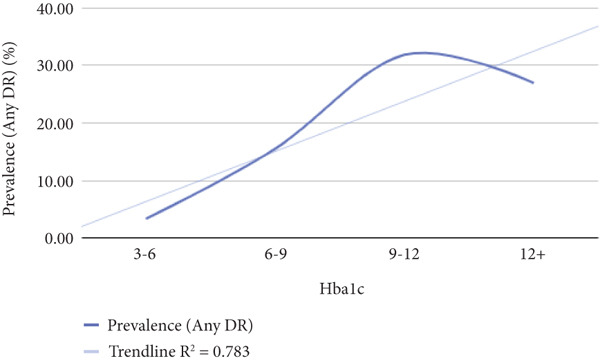
(b)

**Figure:**
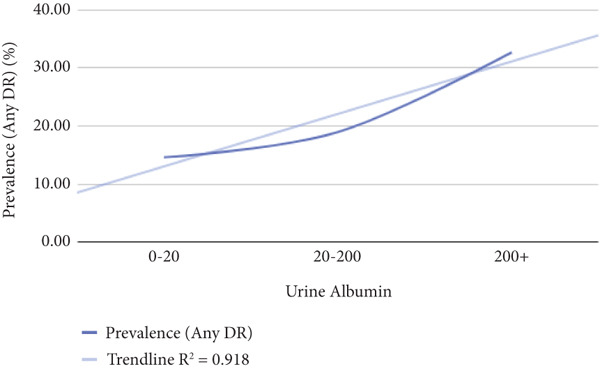
(c)

**Figure:**
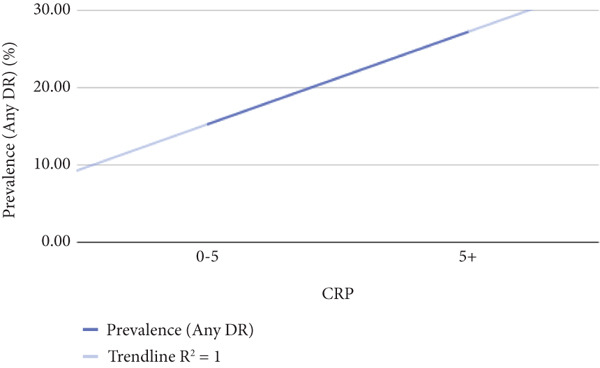
(d)

All other biomarkers, which include LDL cholesterol, HDL cholesterol, GFR, and creatinine levels, were observed to have either no or very weak correlation to DR (Figure 5).

### 3.2. Validation of DR Diagnosis Using AI

The EyeCheckup AI system, when paired with the Canon CR2 AF camera, demonstrated a sensitivity and specificity of 95.65% and 95.92%, respectively, for detecting mtmDR. Similarly, the Topcon TRC‐NW400 achieved 95.19% sensitivity and 96.46% specificity, whereas the Optomed Aurora showed 90.48% sensitivity and 97.21% specificity. For vtDR, the Canon CR2 AF achieved 96.00% sensitivity and 96.34% specificity, the Topcon TRC‐NW400 reached 98.52% sensitivity and 95.93% specificity, and the Optomed Aurora attained 95.12% sensitivity and 98.82% specificity. In the detection of CSDME, the Canon CR2 AF showed 95.83% sensitivity and 96.83% specificity, the Topcon TRC‐NW400 achieved 98.50% sensitivity and 96.52% specificity, and the Optomed Aurora demonstrated 94.93% sensitivity and 98.95% specificity.

In terms of diagnosability, the EyeCheckup software combined with the Optomed, Canon CR2 AF, and Topcon TRC‐NW400 cameras achieved diagnosability rates of 96.57%, 100%, and 100%, respectively (Table 3) [13]. These results highlight the high diagnostic accuracy and reliability of the AI system across different imaging devices [13].

**Table 3:** Performance metrics for diabetic retinopathy screening using nonmydriatic fundus cameras.

**Optomed aurora (** study population=875 **)**			
	**MtmDR**	**VtDR**	**CSDME**

Sensitivity	(114/126)	(78/82)	(75/79)
	90.48%	95.12%	94.93%
	[0.8395–0.9498]	[0.8798–0.9865]	[0.8754–0.9860]
Specificity	(699/719)	(754/763)	(758/766)
	97.21%	98.82%	98.95%
	[0.95737–0.9829]	[0.9777–0.9946]	[0.9795–0.9955]
Accuracy	96.2%	98.4%	98.5%
Diagnosability	845/875		
	96.57%		
Dilation for EyeCheckup usage	526/845		
	62.24%		

**Canon CR2 AF (** study population=704 **)**			
	**MtmDR**	**VtDR**	**CSDME**

Sensitivity	(110/115)	(72/75)	(69/72)
	95.65%	96.00%	95.83%
	[0.9015–0.9857]	[0.8875–0.9917]	[0.8830–0.9913]
Specificity	(565/589)	(606/629)	(612/632)
	95.92%	96.34%	96.83%
	[0.9300–0.9737]	[0.9456–0.9767]	[0.9515–0.9806]
Accuracy	95.8%	96.3%	96.7%
Diagnosability	704/704+		
	100%		
Dilation for EyeCheckup usage	217/704		
	30.82%		

**Topcon NW400 (** study population=585 **)**			
	**MtmDR**	**VtDR**	**CSDME**

Sensitivity	(99/104)	(67/68)	(66/67)
	95.19%	98.52%	98.50%
	[0.8914–0.9842]	[0.9208–0.9996]	[0.9196–0.9996]
Specificity	(464/481)	(496/517)	(499/517)
	96.46%	95.93%	96.52%
	[0.9440–0.9793]	[0.9386–0.9747]	[0.9455–0.9792]
Accuracy	96.2%	96.2%	96.5%
Diagnosability	585/585		
	100%		
Dilation for EyeCheckup usage	459/585		
	78.46%		

## 4. Dıscussıon

There have been previous studies of the relationship between metabolic biomarkers and the prevalence of AI [8, 9], yet there has not been significant implementation in the field of endocrinology to manage or prevent DR using these biomarkers. The data collected from this study provides an interesting case where a model could be developed for predicting the development of DR using the duration of diabetes, urine albumin, CRPs, and HbA1c levels. Certain other biomarkers might also have a significant relationship to the development of DR such as TG, yet more research on a larger population is needed to establish a strong relationship between the prevalence of DR and TG. It is essential to recognize that these biomarkers are not isolated entities. They often work in a complex interplay, with a combination of multiple markers providing a more comprehensive picture of an individual′s DR risk and disease progressiveness. Although not yet routinely used for clinical decision‐making, further research can explore the potential of incorporating these biomarkers into risk stratification models. This holds promise for the following:

1. Early identification of high‐risk individuals, enabling early intervention and potentially preventing vision loss.
2. Personalized treatment approaches, tailoring treatment plans based on individual risk profiles can lead to more effective management strategies.
3. Monitoring treatment efficacy, biomarkers can help assess treatment response and guide adjustments as needed.

The application of AI for DR screening was thoroughly investigated and validated in this study. The long‐term advantages of AI‐based solutions in risk stratification and prognosis for DR screening are evident [14]. As the global burden of vision loss due to DR continues to rise, AI technology has the potential to enhance the efficiency of existing DR teleophthalmology screening programs. By integrating collaborative semiautomated models, AI can support trained human graders and ophthalmologists, enabling a combined approach of human expertise and machine precision [15]. Since the groundbreaking findings of Abramoff et al. in 2008, significant progress has been made in developing automated retinal image analysis systems. These systems leverage deep learning algorithms to accurately detect DR using color digital retinal images [6, 7].

This study, conducted on a large diabetic population without a prior diagnosis of DR, demonstrated that the predefined success thresholds for sensitivity and specificity in grading DR and detecting suspected CSDME were significantly surpassed (85% and 82.5%, respectively). The EyeCheckup AI‐assisted diagnostic system, combined with a comprehensive evaluation of patient metabolic biomarkers, shows great potential for enabling early diagnosis and optimal treatment of DR. By identifying the disease at an early stage, the system can help prevent vision loss caused by DR‐related complications. Additionally, physicians with limited expertise in DR management can use this software to identify patients who require further evaluation and treatment. As a result, the EyeCheckup AI‐assisted diagnostic system holds considerable promise for enhancing the overall quality of care for diabetic patients.

This study boasts several significant strengths. Firstly, it features a large prospective sample size representative of the intended use population. Secondly, it utilized multiple nonmydriatic cameras, including a handheld device, alongside the AI algorithm to diagnose and grade DR and detect suspected CSDME. Furthermore, the AI‐generated diagnoses were validated against assessments made by retinal specialists, ensuring a high level of accuracy and reliability in the results. Importantly, this study was designed to replicate real‐world conditions within an endocrinology clinic. Patients without a prior DR diagnosis were identified and referred to the ophthalmology department for further evaluation and treatment using an AI solution in collaboration with retina specialists.

Wei et al. [16] found no association between macular retinal thickness and HbA1c levels. However, other studies have indicated that both high baseline HbA1c and significant reductions in HbA1c may contribute to increased macular retinal thickness [17, 18]. This discrepancy could be due to the differences in the underlying mechanisms of macular retinal thickening compared with hyperglycemia‐related microangiopathy, or it may involve other unidentified factors. Obesity has also been linked to adverse effects on various eye conditions. A growing body of epidemiological research has highlighted a connection between BMI and DR [19]. Elevated BMI is often associated with hypertension and dyslipidemia, both of which are recognized risk factors for DR [20]. However, our study did not find a significant correlation between BMI and DR. Instead, we observed that DR was positively associated with diabetes duration, total cholesterol (TC), TG, systolic blood pressure, and diastolic blood pressure. These biochemical markers may help predict changes in macular retinal thickness during the early stages of DME.

Numerous studies have examined the prevalence of retinopathy and albuminuria in patients with Type 2 diabetes mellitus (T2DM), reporting DR rates ranging from 16% to 53% [21, 22]. Although microalbuminuria is common in patients without DR (33.9%), the incidence of macroalbuminuria in this group is much lower (4.5%). Parving et al. [23] reported a 22% incidence of microalbuminuria in T2DM patients, whereas Lunetta′s study [24] found a slightly lower rate of 15%. Despite some variations, most studies have demonstrated a clear correlation between the severity of retinopathy and albuminuria. A notable large‐scale–real‐world study by Chavla et al. [25] confirmed significant associations between glycemic control, BMI, diabetes duration, microalbuminuria, peripheral neuropathy, and the severity of retinopathy. The strong link between microalbuminuria and retinopathy in T2DM patients suggests that microalbuminuria could serve as a useful marker for predicting the development of proliferative DR.

In the meta‐analysis conducted by Song et al. [26], CRP levels were found to be higher in individuals with diabetes compared with the control group, and this association was consistent regardless of the type of diabetes. This suggests that CRP levels may be linked to the severity of DR. Similarly, Schram et al. [27] investigated microvascular complications and cardiovascular diseases in Type 1 diabetes and observed statistically significant differences in CRP concentrations among three groups: patients without retinopathy, those with NPDR, and those with PDR. However, these differences disappeared after adjusting for BMI. Izuora et al. [28] reported comparable findings, initially identifying a significant correlation between CRP and retinopathy severity, which lost significance after controlling for age, diabetes duration, gender, and BMI. Mysliwska et al. [29] further demonstrated that CRP levels increased with prolonged diabetes duration, and these changes were statistically significant. Collectively, these findings, particularly from the recent meta‐analysis [26], suggest that CRP levels could serve as a biomarker for assessing DR severity. In our study, a significant correlation between DR and CRP was also observed.

A separate meta‐analysis [30] revealed that lipid levels, including TC, TG, and low‐density lipoprotein cholesterol (LDL‐C), were significantly higher in patients with advanced DR compared with those without DR. However, no significant difference was found in high‐density lipoprotein cholesterol (HDL‐C) levels between the two groups. These results imply that managing serum lipid levels may help prevent DR in patients with T2DM. Takele et al. [31] identified TC levels above 200 mg/dL as a significant predictor of DR, with an adjusted hazard ratio of 2.22 (1.08–4.55). Jin et al. [32], in a large community‐based prospective cohort study, found that low TG levels were independently protective against DR progression in T2DM patients. Another cohort study highlighted LDL‐C levels as a critical factor for DR development in elderly T2DM patients in Taiwan [33]. Additionally, a retrospective cohort study supported the observation that baseline TC, TG, and LDL‐C levels were generally higher in patients with DR compared with those without DR at the time of diabetes diagnosis [34]. Dyslipidemia‐induced upregulation of circulating cytokines, such as VEGF‐A, VEGF‐D, and PlGF, may contribute to the onset and progression of DR [35]. Furthermore, oxidative stress and endoplasmic reticulum stress have been implicated in DR development [36]. Yang et al. [37] suggested that dyslipidemia‐induced mitochondrial damage could accelerate retinal neuron apoptosis, leading to microvascular damage and retinal degeneration in diabetes [38].

A limitation of our study is that suspected cases of CSDME were assessed solely through retinal fundus imaging. Optical coherence tomography (OCT) might have provided more accurate detection of DME. Another limitation is the lack of analysis regarding the history of diabetes medication use, which could influence patient outcomes. Future case‐control studies examining the effects of different antidiabetic drugs on DR could help clarify this issue, and we plan to explore this factor in subsequent research.

Despite the promising potential, several challenges remain. Further research is needed to validate the clinical utility of these metabolic biomarkers and establish standardized reference ranges for their use. Additionally, the cost‐effectiveness and accessibility of biomarker testing must be carefully evaluated to ensure its feasibility for widespread integration into DR management programs.

In summary, the use of biomarkers in DR management offers significant potential for assessing the risk of DR development. As research progresses, these tools could transform the way we prevent, diagnose, and treat DR, ultimately preserving vision and enhancing the quality of life for millions of individuals living with diabetes.

The EyeCheckup AI model, combined with nonmydriatic fundus imaging, has demonstrated high accuracy, sensitivity, and specificity in diagnosing DR and detecting CSDME using fundus photographs captured by various nonmydriatic cameras, as evidenced in this study. The software′s performance closely matched the diagnoses provided by a panel of three retina specialists, which served as the ground truth for this research. This strong performance underscores the model′s reliability as a practical tool for real‐world DR screening, enabling eye care professionals to deliver faster and more accurate diagnoses. Furthermore, studies suggest that the AI′s analysis of two‐field nonmydriatic fundus photographs is on par with the diagnostic accuracy of retina specialists using dilated four wide field or seven standard field images. These findings underscore the potential of AI algorithms to effectively screen diabetic patients who require urgent evaluation and potential treatment by an ophthalmologist, facilitating early intervention and improved patient outcomes.

## 5. Conclusıon

Our study showed a strong association between the patients′ length of diabetes and higher HbA1c, CRP, and urine albumin levels, as well as the prevalence of DR. This suggests that biomarkers may be useful predictors for determining a patient′s risk of developing DR. Clinicians can more precisely assess a patient′s risk profile for DR by comprehending the strong association between elevated HbA1c values, which indicate long‐term blood sugar control, and the length of diabetes. Therefore, incorporating these factors into regular clinical evaluations may improve proactive screening methods, allowing for early identification and treatment to slow the advancement of DR and lower the risk of vision loss in those who are impacted. The duration of DM and poor diabetes control were two factors that showed significant correlations with the prevalence of DR. The prevalence of DR is also thought to be correlated with other factors, such as advanced age, gender, type of DM, smoking, alcohol use, and other disorders like hypertension. The study also shows how early detection of DR can be achieved by combining AI software with nonmydriatic fundus cameras. Every camera that was tested showed varied but sufficient levels of sensitivity and specificity. The results of this study highlight how crucial it is to take into account AI software and a nonmydriatic camera when screening for DR. Last but not the least, laboratory data in endocrinology settings might be used to create screening criteria for risk assessment of DR.

## Ethics Statement

This study was approved by the Ethics Committee of Akdeniz University.

## Conflicts of Interest

The authors declare no conflicts of interest.

## Author Contributions

The clinical study was designed and conceptualized by A.B.B., M.E.D., and M.A. A.B.B., M.E.D., and M.A. played key roles in the study′s design, execution, manuscript revision, and final approval. They also agreed to take responsibility for all aspects of the work. R.S. contributed to the study′s design, drafted and revised the manuscript, approved the final version, and agreed to be accountable for all aspects of the work. Y.A. was involved in the study′s design, conducted the research, revised the manuscript, approved the final version, and agreed to take responsibility for all aspects of the work. M.B. participated in the study′s design, reviewed and revised the manuscript, approved the final version, and agreed to be accountable for all aspects of the work. M.E.D. contributed to the study′s design and conducted the research.

## Funding

This study was supported by the National Institutes of Health of Turkey (TÜSEB).

## Data Availability

The authors can provide data upon reasonable request.

## Appendix

**Table A1:** Determination of sample size.

		**Given H0**	**Given H1**	**Target**	**Actual**		**Reject H0**
Power	N	(P0)	(P1)	Alpha	Alpha	Beta	If Z > =this
0,8033	5341	0,9000	0,9100	0,0500	0,0511	0,1967	1,6449
0,8002	2286	0,9000	0,9150	0,0500	0,0520	0,1998	1,6449
0,8015	1255	0,9000	0,9200	0,0500	0,0525	0,1985	1,6449
0,8004	778	0,9000	0,9250	0,0500	0,0530	0,1996	1,6449
0,8012	523	0,9000	0,9300	0,0500	0,0541	0,1988	1,6449
0,8047	376	0,9000	0,9350	0,0500	0,0545	0,1953	1,6449
0,8092	284	0,9000	0,9400	0,0500	0,0539	0,1908	1,6449
0,8076	212	0,9000	0,9450	0,0500	0,0565	0,1924	1,6449
0,8004	164	0,9000	0,9500	0,0500	0,0553	0,1996	1,6449

## References

[1] Fong D. S. , Aiello L. , Gardner T. W. , King G. L. , Blankenship G. , Cavallerano J. D. , Ferris F. L.3rd, and Klein R. , American Diabetes Association, Diabetic Retinopathy. Diabetes Care. (2003) 26, no. supplement 1, S99–S102, 10.2337/diacare.26.2007.s99, 12502630. 12502630

[2] Abràmoff M. D. , Folk J. C. , Han D. P. , Walker J. D. , Williams D. F. , Russell S. R. , Massin P. , Cochener B. , Gain P. , Tang L. , Lamard M. , Moga D. C. , Quellec G. , and Niemeijer M. , Automated Analysis of Retinal Images for Detection of Referable Diabetic Retinopathy, JAMA Ophthalmology. (2013) 131, no. 3, 351–357, 10.1001/jamaophthalmol.2013.1743, 2-s2.0-84875180389. 23494039

[3] Satman I. , Omer B. , Tutuncu Y. , Kalaca S. , Gedik S. , Dinccag N. , Karsidag K. , Genc S. , Telci A. , Canbaz B. , Turker F. , Yilmaz T. , Cakir B. , Tuomilehto J. , and TURDEP-II Study Group , Twelve-Year Trends in the Prevalence and Risk Factors of Diabetes and Prediabetes in Turkish Adults, European Journal of Epidemiology. (2013) 28, no. 2, 169–180, 10.1007/s10654-013-9771-5, 2-s2.0-84879500031. 23407904 PMC3604592

[4] Fonda S. J. , Bursell S. E. , Lewis D. G. , Clary D. , Shahon D. , and Silva P. S. , Prevalence of Diabetic Eye Diseases in American Indians and Alaska Natives (AI/AN) as Identified by the Indian Health Service′s National Teleophthalmology Program Using Ultrawide Field Imaging (UWFI), Ophthalmic Epidemiology. (2022) 29, no. 6, 672–680, 10.1080/09286586.2021.1996611, 34726132. 34726132 PMC12611973

[5] Scanlon P. H. , The English National Screening Programme for Sight-Threatening Diabetic Retinopathy, Journal of Medical Screening. (2008) 15, no. 1, 1–4, 10.1258/jms.2008.008015, 2-s2.0-43049109886, 18416946. 18416946

[6] Abràmoff M. D. , Lavin P. T. , Birch M. , Shah N. , and Folk J. C. , Pivotal Trial of an Autonomous AI-Based Diagnostic System for Detection of Diabetic Retinopathy in Primary Care Offices, NPJ Digital Medicine. (2018) 1, no. 1, 10.1038/s41746-018-0040-6. PMC6550188 31304320

[7] Grzybowski A. , Brona P. , Lim G. , Ruamviboonsuk P. , Tan G. S. W. , Abramoff M. , and Ting D. S. W. , Artificial Intelligence for Diabetic Retinopathy Screening: A Review, Eye. (2020) 34, no. 3, 451–460, 10.1038/s41433-019-0566-0, 2-s2.0-85073776664. 31488886 PMC7055592

[8] Hou X. W. , Wang Y. , and Pan C. W. , Metabolomics in Diabetic Retinopathy: A Systematic Review, Investigative Ophthalmology & Visual Science. (2021) 62, no. 10, 10.1167/iovs.62.10.4. PMC8340662 34347011

[9] Jenkins A. J. , Joglekar M. V. , Hardikar A. A. , Keech A. C. , O′Neal D. N. , and Januszewski A. S. , Biomarkers in Diabetic Retinopathy, Review of Diabetic Studies. (2015) 12, no. 1-2, 159–195, 10.1900/RDS.2015.12.159, 2-s2.0-84982728059, 26676667. PMC5397989 26676667

[10] Flaxel C. J. , Adelman R. A. , Bailey S. T. , Fawzi A. , Lim J. I. , Vemulakonda G. A. , and Ying G. S. , Diabetic Retinopathy Preferred Practice Pattern®, Ophthalmology. (2020) 127, no. 1, P66–P145, 10.1016/j.ophtha.2019.09.025, 31757498. 31757498

[11] Laursen M. L. , Moeller F. , Sander B. , and Sjoelie A. K. , Subthreshold Micropulse Diode Laser Treatment in Diabetic Macular Oedema, British Journal of Ophthalmology. (2004) 88, no. 9, 1173–1179, 10.1136/bjo.2003.040949, 2-s2.0-4344693990, 15317711. 15317711 PMC1772323

[12] Lim J. I. , Kim S. J. , Bailey S. T. , Kovach J. L. , Vemulakonda G. A. , Ying J. S. , and Flaxe C. J. , American Academy of Ophthalmology Preferred Practice Pattern Retina/Vitreous Committee. Diabetic Retinopathy Preferred Practice Pattern®, Ophthalmology. (2025) 127, no. 1, P75–P162, 10.1016/j.ophtha.2024.12.020, 39918521. 39918521

[13] Doğan M. E. , Bilgin A. B. , Sari R. , Bulut M. , Akar Y. , and Aydemir M. , Head to Head Comparison of Diagnostic Performance of Three Non-Mydriatic Cameras for Diabetic Retinopathy Screening With Artificial Intelligence, Eye. (2024) 38, no. 9, 1694–1701, 10.1038/s41433-024-03000-9, 38467864. 38467864 PMC11156854

[14] Gunasekeran D. V. , Ting D. S. W. , Tan G. S. W. , and Wong T. Y. , Artificial Intelligence for Diabetic Retinopathy Screening, Prediction and Management, Current Opinion in Ophthalmology. (2020) 31, no. 5, 357–365, 10.1097/ICU.0000000000000693, 32740069. 32740069

[15] Ting D. S. W. , Cheung C. Y. , Lim G. , Tan G. S. W. , Quang N. D. , Gan A. , Hamzah H. , Garcia-Franco R. , San Yeo I. Y. , Lee S. Y. , Wong E. Y. M. , Sabanayagam C. , Baskaran M. , Ibrahim F. , Tan N. C. , Finkelstein E. A. , Lamoureux E. L. , Wong I. Y. , Bressler N. M. , Sivaprasad S. , Varma R. , Jonas J. B. , He M. G. , Cheng C. Y. , Cheung G. C. M. , Aung T. , Hsu W. , Lee M. L. , and Wong T. Y. , Development and Validation of a Deep Learning System for Diabetic Retinopathy and Related Eye Diseases Using Retinal Images From Multiethnic Populations With Diabetes, Journal of the American Medical Association. (2017) 318, no. 22, 2211–2223, 10.1001/jama.2017.18152, 2-s2.0-85038438910, 29234807. 29234807 PMC5820739

[16] Wei Q. , Qiu W. , Liu Q. , and Jiang Y. , Relationship Between Risk Factors and Macular Thickness in Patients With Early Diabetic Retinopathy, International Journal of General Medicine. (2022) 15, no. 2022, 6021–6029, 10.2147/IJGM.S366348, 35818578. 35818578 PMC9270927

[17] Moon S. W. , Kim H. Y. , Kim S. W. , Oh J. , Huh K. , and Oh I. K. , The Change of Macular Thickness Measured by Optical Coherence Tomography in Relation to Glycemic Control in Diabetic Patients, Graefe′s Archive for Clinical and Experimental Ophthalmology. (2011) 249, no. 6, 839–848, 10.1007/s00417-010-1562-z, 2-s2.0-79959718699, 21110036. 21110036

[18] Yeung L. , Sun C. C. , Ku W. C. , Chuang L. H. , Chen C. H. , Huang B. Y. , Ting M. K. , and Yang K. J. , Associations Between Chronic Glycosylated Haemoglobin (Hba1c) Level and Macular Volume in Diabetes Patients Without Macular Oedema, Acta Ophthalmologica. (2010) 88, no. 7, 753–758, 10.1111/j.1755-3768.2009.01711.x, 2-s2.0-78349269558, 19878106. 19878106

[19] Kaštelan S. , Salopek Rabatić J. , Tomić M. , Gverović Antunica A. , Ljubić S. , Kaštelan H. , Novak B. , and Orešković D. , Body Mass Index and Retinopathy in Type 1 Diabetic Patients, International Journal of Endocrinology. (2014) 2014, no. 1, 387919, 10.1155/2014/387919, 2-s2.0-84897759934, 24696683. 24696683 PMC3948586

[20] Yau J. W. , Rogers S. L. , Kawasaki R. , Lamoureux E. L. , Kowalski J. W. , Bek T. , Chen S. J. , Dekker J. M. , Fletcher A. , Grauslund J. , Haffner S. , Hamman R. F. , Ikram M. K. , Kayama T. , Klein B. E. , Klein R. , Krishnaiah S. , Mayurasakorn K. , O′Hare J. P. , Orchard T. J. , Porta M. , Rema M. , Roy M. S. , Sharma T. , Shaw J. , Taylor H. , Tielsch J. M. , Varma R. , Wang J. J. , Wang N. , West S. , Xu L. , Yasuda M. , Zhang X. , Mitchell P. , and Wong T. Y. , Global Prevalence and Major Risk Factors of Diabetic Retinopathy, Diabetes Care. (2012) 35, no. 3, 556–564, 10.2337/dc11-1909, 2-s2.0-84859030420, 22301125. 22301125 PMC3322721

[21] Eggertsen R. , Kalm H. , and Blohmé G. , The Value of Screening for Retinopathy and Microalbuminuria in Patients With Type 2 Diabetes in Primary Health Care, Scandinavian Journal of Primary Health Care. (1993) 11, no. 2, 135–140, 10.3109/02813439308994916, 2-s2.0-0027284431, 8356364. 8356364

[22] Parving H. H. , Hommel E. , Mathiesen E. , Skøtt P. , Edsberg B. , Bahnsen M. , Lauritzen M. , Hougaard P. , and Lauritzen E. , Prevalence of Microalbuminuria, Arterial Hypertension, Retinopathy and Neuropathy in Patients With Insulin Dependent Diabetes, British Medical Journal (Clinical Research Edition). (1988) 296, no. 6616, 156–160, 10.1136/bmj.296.6616.156, 2-s2.0-0024289644, 3122980. PMC2544895 3122980

[23] Sobngwi E. , Mbanya J. C. , Moukouri E. N. , and Ngu K. B. , Microalbuminuria and Retinopathy in a Diabetic Population of Cameroon, Diabetes Research and Clinical Practice. (1999) 44, no. 3, 191–196, 10.1016/s0168-8227(99)00052-2, 2-s2.0-0033036091, 10462142. 10462142

[24] Lunetta M. , Infantone L. , Calogero A. E. , and Infantone E. , Increased Urinary Albumin Excretion is a Marker of Risk for Retinopathy and Coronary Heart Disease in Patients With Type 2 Diabetes Mellitus, Diabetes Research and Clinical Practice. (1998) 40, no. 1, 45–51, 10.1016/s0168-8227(98)00024-2, 2-s2.0-0031816744, 9699090. 9699090

[25] Chawla S. , Trehan S. , Chawla A. , Jaggi S. , Chawla R. , Kumar V. , and Singh D. , Relationship Between Diabetic Retinopathy Microalbuminuria and Other Modifiable Risk Factors, Primary Care Diabetes. (2021) 15, no. 3, 567–570, 10.1016/j.pcd.2021.01.012, 33551334. 33551334

[26] Song J. , Chen S. , Liu X. , Duan H. , Kong J. , and Li Z. , Relationship Between C-Reactive Protein Level and Diabetic Retinopathy: A Systematic Review and Meta-Analysis, PLoS One. (2015) 10, no. 12, e0144406, 10.1371/journal.pone.0144406, 2-s2.0-84955499663, 26636823. 26636823 PMC4670229

[27] Schram M. T. , Chaturvedi N. , Schalkwijk C. G. , Fuller J. H. , Stehouwer C. D. , and EURODIAB Prospective Complications Study Group , Markers of Inflammation are Cross-Sectionally Associated With Microvascular Complications and Cardiovascular Disease in Type 1 diabetes?the EURODIAB Prospective Complications Study, Diabetologia. (2005) 48, no. 2, 370–378, 10.1007/s00125-004-1628-8, 2-s2.0-17444405235, 15692810. 15692810

[28] Izuora K. E. , Chase H. P. , Jackson W. E. , Coll J. R. , Osberg I. M. , Gottlieb P. A. , Rewers M. J. , and Garg S. K. , Inflammatory Markers and Diabetic Retinopathy in Type 1 Diabetes, Diabetes Care. (2005) 28, no. 3, 714–716, 10.2337/diacare.28.3.714, 2-s2.0-14644420204. 15735215

[29] Myśliwska J. , Smardzewski M. , Marek-Trzonkowska N. , Myśliwiec M. , and Raczyńska K. , Expansion of CD14+CD16+ Monocytes Producing TNF-*α* in Complication-Free Diabetes Type 1 Juvenile Onset Patients, Cytokine. (2012) 60, no. 1, 309–317, 10.1016/j.cyto.2012.03.010, 2-s2.0-84865415513, 22484242. 22484242

[30] Li Z. , Yuan Y. , Qi Q. , Wang Q. , and Feng L. , Relationship Between Dyslipidemia and Diabetic Retinopathy in Patients With Type 2 Diabetes Mellitus: A Systematic Review and Meta-Analysis, Systematic Reviews. (2023) 12, no. 1, 10.1186/s13643-023-02321-2, 37620980. PMC10463379 37620980

[31] Takele M. B. , Boneya D. J. , Alemu H. A. , Tsegaye T. B. , Birhanu M. Y. , Alemu S. , and Anto T. G. , Retinopathy Among Adult Diabetics and Its Predictors in Northwest Ethiopia, Journal Diabetes Research. (2022) 2022, no. 1, 1362144, 10.1155/2022/1362144. PMC8863468 35211627

[32] Jin P. , Peng J. , Zou H. , Wang W. , Fu J. , Shen B. , Bai X. , Xu X. , and Zhang X. , The 5-Year Onset and Regression of Diabetic Retinopathy in Chinese Type 2 Diabetes Patients, PLoS One. (2014) 9, no. 11, e113359, 10.1371/journal.pone.0113359, 2-s2.0-84913589661, 25402474. 25402474 PMC4234658

[33] Chiu T. T. , Tsai T. L. , Su M. Y. , Yang T. , Tseng P. L. , Lee Y. J. , and Lee C. H. , The Related Risk Factors of Diabetic Retinopathy in Elderly Patients With Type 2 Diabetes Mellitus: A Hospital-Based Cohort Study in Taiwan, International Journal of Environmental Research and Public Health. (2021) 18, no. 1, 10.3390/ijerph18010307, 33406594. PMC7795133 33406594

[34] Debele G. R. , Kanfe S. G. , Weldesenbet A. B. , Ayana G. M. , Jifar W. W. , and Raru T. B. , Incidence of Diabetic Retinopathy and Its Predictors Among Newly Diagnosed Type 1 and Type 2 Diabetic Patients: A Retrospective Follow-Up Study at Tertiary Health-Care Setting of Ethiopia, Diabetes, Metabolic Syndrome and Obesity. (2021) 14, 1305–1313, 10.2147/DMSO.S300373, 33790598. PMC7997545 33790598

[35] Zhang X. , Qiu B. , Wang Q. , Sivaprasad S. , Wang Y. , Zhao L. , Xie R. , Li L. , and Kang W. , Dysregulated Serum Lipid Metabolism Promotes the Occurrence and Development of Diabetic Retinopathy Associated With Upregulated Circulating Levels of VEGF-A, VEGF-D, and PlGF, Frontiers in Medicine. (2021) 8, 779413, 10.3389/fmed.2021.779413, 34904074. 34904074 PMC8664628

[36] Rao H. , Jalali J. A. , Johnston T. P. , and Koulen P. , Emerging Roles of Dyslipidemia and Hyperglycemia in Diabetic Retinopathy: Molecular Mechanisms and Clinical Perspectives, Frontiers in endocrinology. (2021) 12, 620045, 10.3389/fendo.2021.620045, 33828528. 33828528 PMC8020813

[37] Yang C. , Xie L. , Gu Q. , Qiu Q. , Wu X. , and Yin L. , 7-Ketocholesterol Disturbs RPE Cells Phagocytosis of the Outer Segment of Photoreceptor and Induces Inflammation Through ERK Signaling Pathway, Experimental Eye Research. (2019) 189, 107849, 10.1016/j.exer.2019.107849, 2-s2.0-85073816562, 31655042. 31655042

[38] Bonora B. M. , Albiero M. , Morieri M. L. , Cappellari R. , Amendolagine F. I. , Mazzucato M. , Zambon A. , Iori E. , Avogaro A. , and Fadini G. P. , Fenofibrate Increases Circulating Haematopoietic Stem Cells in People With Diabetic Retinopathy: A Randomised, Placebo-Controlled Trial, Diabetologia. (2021) 64, no. 10, 2334–2344, 10.1007/s00125-021-05532-1, 34368894. 34368894

